# Analysis of Fourteen New Cases of Meningovascular Syphilis: Renewed Interest in an Old Problem

**DOI:** 10.7759/cureus.16951

**Published:** 2021-08-06

**Authors:** Faiza Aziouaz, Fatima Zahra Mabrouki, Mohammed Chraa, Nisrine Louhab, Nawal Adali, Imane Hajjaj, Najib Kissani, Yassine Mebrouk

**Affiliations:** 1 Neurology, Faculty of Medicine and Pharmacy, Mohammed VI University Hospital, Oujda, MAR; 2 Ophthalmology, Faculty of Medicine and Pharmacy, Mohammed VI University Hospital, Oujda, MAR; 3 Neurology, Faculty of Medicine and Pharmacy, Mohammed VI University Hospital, Marrakech, MAR

**Keywords:** neurosyphilis, stroke, vasculitis, csf, acquired immune deficiency syndrome (aids)

## Abstract

Neurosyphilis (NS) remains a public health problem. Several recent reports suggest a worldwide increase in the incidence of this condition. Various syndromes can occur in NS, such as syphilitic meningitis, meningovascular syphilis, parenchymatous and gummatous neurosyphilis. Syphilis meningovascular will be the focus of this study. We report 14 new observations of meningovascular syphilis. A review of demographic and clinical features, neuroimaging findings, cerebrospinal fluid changes, treatment and outcome, pathophysiology mechanism of meningovascular syphilis are presented.

## Introduction

The incidence of stroke is approximately 2.3/1000/year, based on community surveys [1]. Stroke can be a complication of a central nervous system infection [2]. Some infections are more often associated with cerebrovascular complications than others, and the pathogenesis of vascular lesions varies widely from one disease to another [2, 3]. Most of these conditions cause stroke through a mechanism of angitis [4]. This review focuses on meningovascular syphilis as an infectious cause of stroke.

Neurosyphilis (NS) management has been proved as being controversial and continues to be debated. It is an infection of the central nervous system caused by the spirochaete Treponema pallidum. The frequent fluctuations of primary and secondary syphilis incidence and Acquired Immunodeficiency Syndrome (AIDS) pandemia have brought NS to the centre of attention in global health [5,6]. The World Health Organization estimate 12 million new cases of Syphilis infection in 1999 [7], and several case studies have also reported changes in the clinical profile of NS, with more severe and earlier clinical manifestations and frequent treatment failures [8,9].

This study was a hospital-based study conducted in a tertiary hospital located in Marrakesh, an area with a relatively high incidence of neurosyphilis, and covers the southern area of Morocco. This study aimed to report and describe the clinical, radiologic and laboratory studies of fourteen cases of meningovascular syphilis in patients with HIV-negative. A review of demographic and clinical features, neuroimaging findings, cerebrospinal fluid changes, treatment and outcome, pathophysiology mechanism of meningovascular syphilis are presented.

## Materials and methods

The medical files of fourteen patients diagnosed with meningovascular syphilis over six years (January 2008-December 2014) at Ibn Tofail hospital, University Hospital Mohammed VI-Marrakech (the only institution for referral of neurological diseases in the South of Morocco), were retrospectively reviewed. The presence of positive treponemal tests in blood, especially Venereal Disease Research Laboratory (VDRL) test and Reactive Treponema Pallidum Particle Agglutination (TPPA) test, or VDRL positive cerebrospinal fluid in the absence of gross blood contamination allow the diagnosis to be established. CT was available for all patients; MRI for three patients and one patient was studied by MR angiography. The leukocyte count above 5 cells/mm3 and proteinorrachia above 40mg/dl at the lumbar level where cerebrospinal fluid (CSF) parameters are considered to be abnormal. For 10 days and then every three months for one year, all patients received high-dose intravenous penicillin: crystalline penicillin G (30 million U IV daily) for 10 days, 3-monthly for one year. In order to assess the response to therapy, treatment failure was defined by any of the following: clinical deterioration, the emergence of new symptoms, increasing or persistent CSF pleocytosis at six months and increasing or persistent VDRL titers at six months. The average follows up was 21 months of all patients.

## Results

All patients were over 30 years old and fulfilling the criteria for NS. Twelve were men, and two were female. The mean age was 48 years with a range from 31 to 60 years. Extramarital and unprotected extramarital sexual intercourse was reported in 5 patients (36%). All patients tested HIV-seronegative. Clinical, biological, cardiovascular, ophthalmologic and electroencephalographic findings were summarized in tables 1, 2.

**Table 1:** Clinical, cardiovascular, ophthalmologic and EEG data from 14 patients with meningovascular syphilis. PLED- Periodic Lateralized Epileptiform Discharges; EEG- Electroencephalogram

Pt. No	Age (year)	SEX	Extramarital, unprotected sex	cardiovascular risk factors	Prodrome clinical course		Symptoms	Neurological signs	ECG and echocardiography	Vascular Doppler	Ocular syphilis	EEG findings	F-up (months)
					SYMPTOMS	DURATION							
1	52	M	No	Cigarette smoking	*Memory loss* *Persistent headaches*	Six months	*Acute onset of expressiveaphasia*	*Normalcranial nerves with no motor or sensorydeficits*	Unremarkable	Unremarkable	Unremarkable	Normal	13
2	38	M	Yes	*Cigarette smoking and diabetes mellitus*	*Persistent headaches andIrritability*	Five months	Acute onset of weakness in the right upper and lower limbs	Right hemiparesis and mild motor dysphasia	Unremarkable	Unremarkable	Unremarkable	PLED	7
3	50	M	No	*Elevated blood pressure and cigarette smoking*	*Unremarkable*		*Sudden onset of left-sided hemiparesis*	Left hemiparesis	*Hypertrophic cardiomyopathy* *mitral and aortic valve insufficiency*	Bilateral thrombosis of the internal carotid artery	Iritis, chorioretintis	Normal	23
4	31	M	Yes	Unremarkable	*Vertigo andemotional liability*	One year	Sudden onset of right-sided weakness and inability to talk	Expressive aphasia, right hemiparesis	Unremarkable	Unremarkable	Unremarkable	Not performed	36
5	43	M	Yes	*Cigarette smoking*	*Unremarkable*		*Acute onset of right-sided hemiparesis.*	Right hemiparesis and mild motor dysphasia	Unremarkable	Unremarkable	Unremarkable	Normal	17
6	51	F	No	Unremarkable	*Insomnia,Irritability andAnxiety*	Not precise	*Acute onset of left-sided hemiparesis.*	*Left-sidedhemiplegia and facial palsy with minor dysarthria*	Mitral and aortic valve insufficiency	Unremarkable	Iritis and vitritis	Normal	23
7	40	M	Yes	*Unremarkable*	Persistent headaches	Two years	Sudden-onset of short-term confusional state	Confusional state, no focal neurological signs were noted	Unremarkable	Unremarkable	Unremarkable	Normal	9
8	45	M	No	*Unremarkable*	Unremarkable		Sudden onset of weakness in the left upper and lower limbs	Left hemiparesis	*Hypertrophic cardiomyopathy*	Thrombosis of the left internal carotid artery	Unremarkable	PLED	27
9	38	M	No	*Unremarkable*	Unremarkable		Sudden loss of vision in his left eye of one-week duration and no other complaints	*Left hemiparesis,visual acuity was 8/10 in the left eye and light perception in the right. Systemic examination was normal.*	Unremarkable	Unremarkable	Right optic atrophy	Normal	28
10	56	M	No	*Unremarkable*	Unremarkable		*Acute onset of * *Hallucinatory state*	*Hallucinatory state* *, no focal neurological signs *	Unremarkable	Unremarkable	Unremarkable	PLED	30
11	60	M	Yes	*Cigarette smoking and diabetes mellitus*	*Personality changes headaches and* *Concentration difficulty*	One-year	Sudden loss of consciousness	*Normal cranial nerves with no motor or sensorydeficits*	*Hypertrophic cardiomyopathy* *mitral and aortic valve insufficiency*	Unremarkable	Unremarkable	Normal	10
12	60	M	No	Elevated blood pressure	*Persistent headaches,vertigo and Irritability*	Seven months	Sudden weakness of the left side	*Right hemianopsia, mild dysrathria, mild weakness and sensory loss on the left side and sensory extinction on the left side*	*Hypertrophic cardiomyopathy* *aortic insufficiency*	Unremarkable	Unremarkable	Not performed	37
13	57	F	No	Diabetes mellitus and elevated blood pressure	Persistent headaches and vertigo,	Two years	*She suddenly became unable to walk or speak*	Right hemiparesis and mild motor dysphasia	*Hypertrophic cardiomyopathy* *aortic insufficiency*	Unilateral thrombosis of the left internal carotid artery	chorioretinitis	Normal	21
14	53	M	No	Diabetes mellitus	*Emotional liability,persistent headaches andconcentration difficulty*	One-year	Sudden onset of mental impairment and Temporary confusion	No focal neurological signs were noted	*Hypertrophic cardiomyopathy*	Unremarkable	Unremarkable	PLED	11

**Table 2:** Cell count, protein, and serological tests in CSF samples from 14 patients with meningovascular syphilis. VDRL- venereal disease research laboratory; FTA-Abs- fluorescent treponemal antibody absorption;

CELL count	PROTEIN	VDRL	FTA-Abs	NUMBER
Normal	Normal	+	Not performed	8
High	Normal	+	Not performed	1
High	Hight	+	Not performed	3
High	Hight	-	+	2

The sudden focal neurologic deficit, without any other sign of encephalopathy or Central nervous system (CNS) infection, simulating a typical stroke presentation, was reported in 9 patients (64%). Altered mental status was seen in three patients (21%) and psychiatric presentation in 1 patient (7%) (patient 10). Finally, a neuroretinitis presentation was noted in one patient (7%) (patient 9). The prodromal clinical course of several months to years before a focal neurologic deficit has been described in 10 patients (71%), such as persistent headaches (70%), and personality changes (60%). Computerized tomography of the brain, MRI and MR angiography findings are summarized in table 3.

**Table 3:** CT, MRI, and angiography findings from 14 patients with meningovascular syphilis.

Findings	NO. (%) of patients
Normal	1 (7%)
Cerebral infarction	
*Cortical/subcortical	7 (50 %)
*Basal ganglia/thalamus	2 (14 %)
*Brain stem	1 (7 %)
Atrophy	
*Mild	4 (28 %)
*Moderate	3 (21 %)
*Severe	2 (15 %)
White matter lesion	6 (42 %)
Arteritis	0 (0%)
Meningeal enhancement	1 (7%)

**Figure 1:**
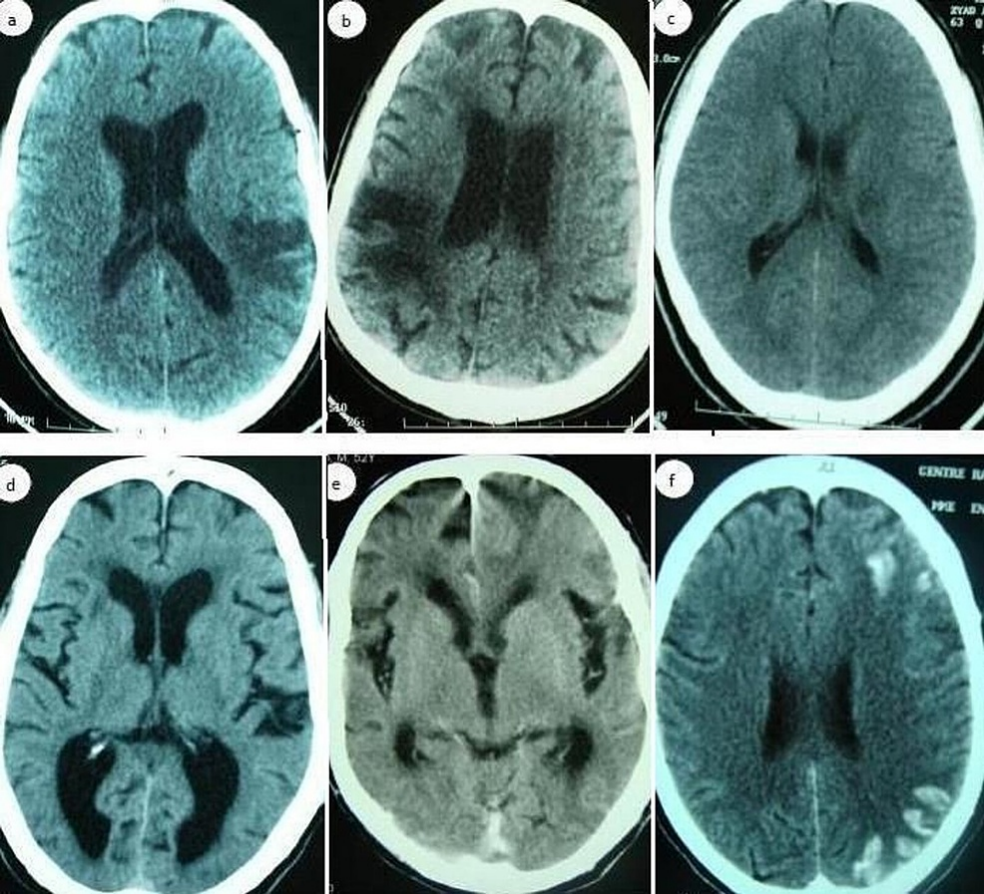
Axial view CT scans; a (patient 2), b (patient 3), note a moderate-to-large well-defined lesion involving white matter and adjacent cortex, conforming to a vascular distribution; c (patient 8), note a focal ischemic lesions in deep area. D (patient 11), e (patient 7), Note a diffuse cerebral atrophy; f (patient 13), note the Meningeal enhancement.

The CT scan was available in all the patients. Ten patients (71.5%) had moderate-to-large well-defined lesions involving white matter and adjacent cortex, conforming to a vascular distribution (fig 1a, fig 1b). Four patients (28.5%) had focal ischemic lesions in the deep area (fig 1c). Diffuse cerebral atrophy was noted in 9 patients (64%) (fig 1d, fig 1e). Meningeal enhancement was only observed in one patient (patient 13) (fig. 1f).

**Figure 2:**
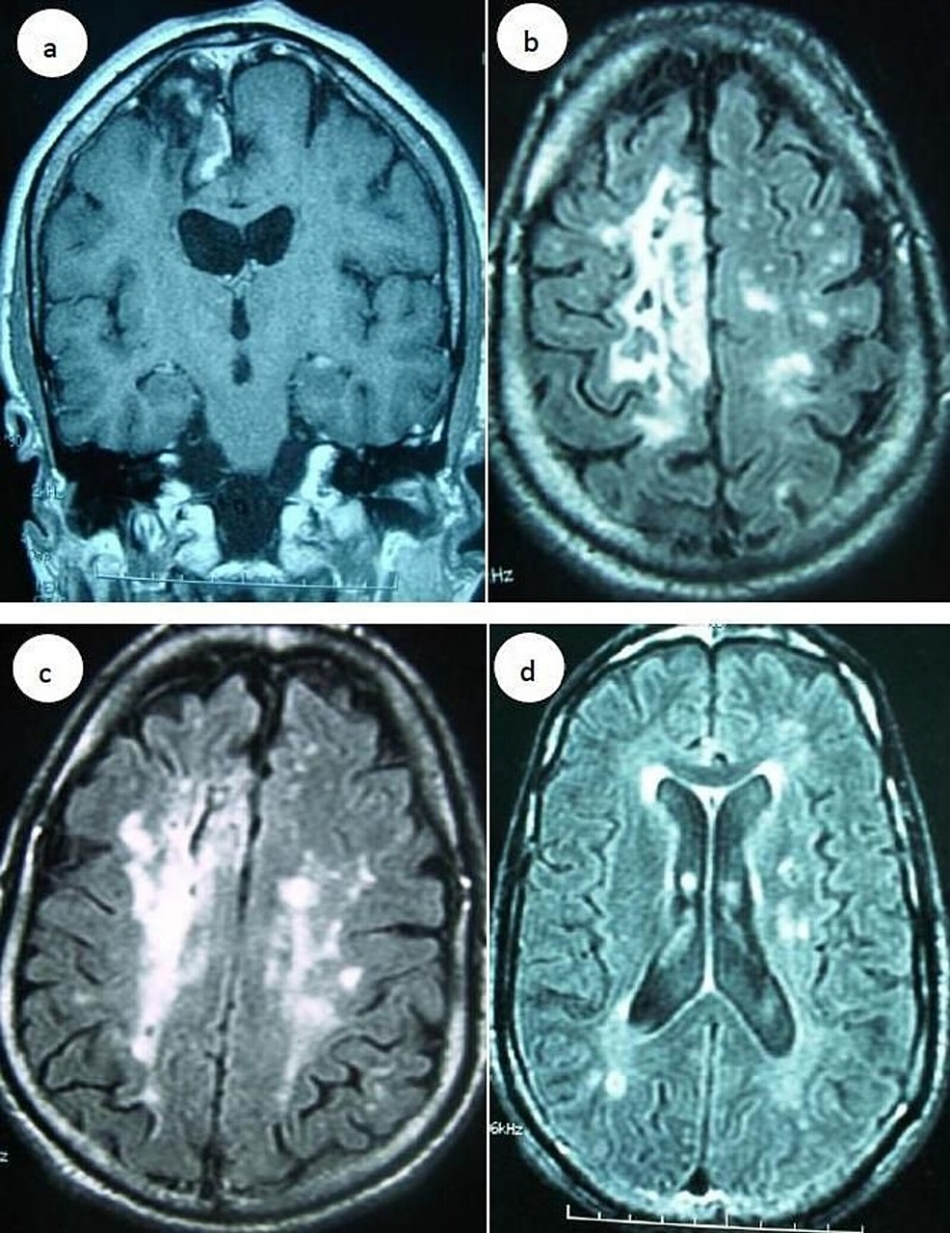
a; brain MRI images coronal T1 demonstrated sequellar areas of infraction; b, c, d, brain MRI images axial T2 showed multiple cerebral infarctions involving one lobe, corona radiate (patient 14) .

MR images in two patients showed multiple cerebral infarctions involving one lobe, corona radiata (patients 3 and 14), and were normal in one patient (patient number 9) (fig. 2). MR angiography performed in one case was normal.

**Figure 3:**
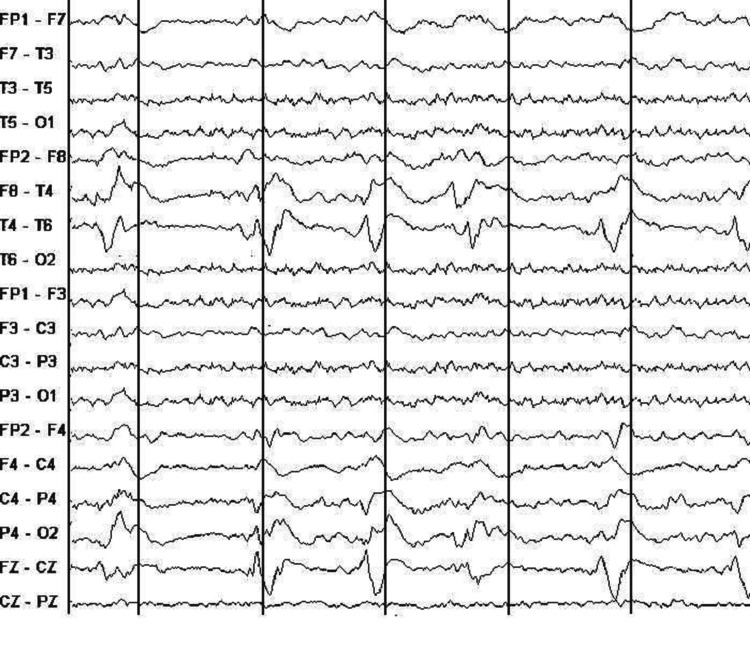
Routine 16 channel scalp EEG demonstrated periodic lateralized epileptiform discharges (PLEDs) (patient 13).

Routine 16 channel scalp electroencephalogram (EEG) was normal in 10 patients (83%) and showed periodic lateralized epileptiform discharges in 2 patients (17%) (fig. 3). The generalized periodic activity wasn’t observed in any patient. Outcome data was available on all patients. The definition of improvement was based on characteristics from the medical register and sequential titer reduction of VDRL. The average follows up was 21 months. Five patients (36%) were noted to have fully recovered. In 7 patients (50%), the recovery was partial. We registered 2 cases of therapeutic failure defined by clinical deterioration.

## Discussion

Syphilis is a sexually transmitted infection caused by the spirochaete Treponema pallidum. It has been a significant cause of neurological impairment for 500 years [10]. The use of penicillin treatment has significantly reduced the incidence of syphilis [5,6]. In recent years, the human immunodeficiency virus (HIV) pandemic has been accompanied by an increasing incidence of syphilis [11]. The World Health Organization estimates 12 million new cases of Syphilis infection in 1999 [7]. The interaction of syphilis and HIV has brought syphilis back to the centre of attention in global health and has stimulated renewed interest in this ancient disease.

NS occurs with CNS invasion, at any stage of syphilis infection, in about 5% to 10% of untreated cases [12]. CNS involvement in syphilis patients is classified into four syndromes based on the clinical symptoms and the time interval between primary infection and the appearance of symptoms: syphilitic meningitis, meningovascular syphilis, parenchymatous and gummatous neurosyphilis [13]. In Morocco, NS remains a significant health problem, particularly in the South. This is most likely due to socioeconomic factors such as limited access to health services leading to under-diagnosis and treatment of latent syphilis [14]. Other contributing factors include low educational levels and the growing sex trade in cities with high tourist activities such as Marrakech and Agadir.

We adopted strict diagnostic criteria of NS in the present investigation. The presence of positive treponemal tests in the blood Venereal Disease Research Laboratory (VDRL) test and Reactive Treponema Pallidum Particle Agglutination (TPPA) test), or VDRL positive cerebrospinal fluid in the absence of gross blood contamination allows the diagnosis to be established. Currently, diagnosis of neurosyphilis must be made with both a careful clinical and laboratory assessment because the polymorphic clinical manifestations, the specificity and sensitivity of supportive laboratory tests are unclear [15]. In patients with HIV infection, this is even more complex [16]. Positive VDRL in the CSF is sufficient to diagnose neurosyphilis in the absence of serum contamination because CSF-VDRL is highly specific. However, a negative VDRL does not eliminate the possibility of the disease because its sensitivity is estimated at 30-70% [17,18]. The only serological evidence of neurosyphilis is CSF fluorescent treponemal antibody absorption (FTA-AB) and carries the advantage of being highly sensitive, but its specificity is low. The primary value of the FTA test in CSF is negative. It will exclude the possibility of neurosyphilis. A positive FTA-ABS in an appropriate clinical setting associated with raised CSF cell count and protein or IgG index, when the CSF VDRL is negative, is a useful method of identifying neurosyphilis [18-20].

The frequency of vascular NS is variable. It is observed in 10% to 35% of all patients with NS in the international literature [21]. In our series (not yet published), meningovascular syphilis accounts for 10% of all cases of NS. Yahyaoui, in his largest NS, meningovascular syphilis represented 21% of all NS cases [14]. Low frequencies, below 15%, have been found in several studies: Hooshmand (11%) [22], Timmermans (14.9%) [23]. Meningovascular syphilis is observed between 4 and 10 years, with a peak of seven years after primary infection, but it may occur as early as six months after the primary infection [21]. Diagnosis is difficult because the clinical manifestations are polymorphic [13].

Even so, a high index of suspicion is necessary to make the diagnosis. Doctors managing stroke patients should be aware of early systemic features of syphilis, such as previous chancre, alopecia, regional lymphadenopathy, retinitis, uveitis and rash. In many previous case reports of meningovascular syphilis, a description of a prodromal clinical course several weeks to months before a focal neurologic deficit has been described. It may include persistent headaches, personality disturbances, emotional liability, vertigo, and insomnia [24]. Data of this study are no exception: seven patients (70%) had migrainous associated symptoms, personality changes were noted in six patients (60%). Clinical manifestations depend on the area of the spinal cord or brain that is affected. It can involve a sudden focal neurologic deficit, without any other sign of Central nervous system (CNS) infection or encephalopathy [13,24-26]. The spectrum of neurological manifestations includes aphasia, hemiparesis, hemianesthesia, meningoradicular irritation (headache and vomiting), diplopia, vertigo, dysarthria, various diffuse encephalic symptoms (apathy, vertigo, and bad attention), and a variety of brainstem syndromes [21,26]. In this series, hemiparesis was seen in 57% of all patients, aphasia was noted in 36% of all patients. Ocular abnormalities in meningovascular syphilis are optic neuritis, iritis and chorioretinitis [27]. One case (patient 9) of syphilitic optic neuritis had presented three months before a probable history of transient ischaemic stroke was found. The patient presented with a 7-day history of sudden painless decreased visual acuity in the right eye. He had no other ocular or systemic complaints on examination, light perception in the right eye and 8/10 in the left. The anterior segment was normal. Retina examination showed bilateral optic atrophy, much more marked in the right eye. Visual evoked potential showed bilateral axonal optic neuritis. MRI did not show any signal abnormality. The CSF was positive for VDRL.

Meningovascular complications of syphilis are related to focal syphilitic endarteritis. It is characterized by inflammatory changes and fibrous in the adventitia, along with a fibroblastic proliferation of the intima and thinning of the media, leading to cerebrovascular thrombosis and ischemic infarction [21,28,29]. The others mechanisms are specific granulomatous infiltration and meningeal inflammatory process [21]. Additionally, a cerebral aneurysm secondary to syphilitic vasculopathy has been reported once in a patient with an aneurysm of the posterior communicating artery [30]. The middle cerebral artery is most often affected, but other intracranial arteries and the anterior spinal artery may also be damaged [31]. The size and location of the infarction are predictive focal neurological deficits [13,24,25,31]. Also, severe vasculopathy is more developed in syphilitic patients coinfected with HIV than HIV-negative patients [32]. 

Syphilitic meningovascular lesions include cortical or subcortical infarction, leptomeningeal enhancement, meningitis and arthritis. Cerebral infarctions can be detected both on CT and MRI as small deep focal lesions. In contrast to atherosclerotic cerebrovascular disease, cerebral infractions are often very discrete due to the involvement of an isolated artery [28]. The infarct area includes corona radiata, the lobe of the brain, basal ganglia/thalamus, cerebellum, brain stem, cingulated and gyrus corpus callosum. Multiple cerebral infarctions have been previously reported [28,33,34]. In this study, 71% of the patients had cerebral infarction, which was located in a deep area in 28% of all patients, and were in multiple territories in two patients.

Other manifestations of meningovascular syphilis include leptomeningeal enhancement, meningitis, leptomeningeal granulomas, gummata (enhancing nodules), meningoneuritis with cranial neuropathies, gummatous periostitis and periostitis involving the otic capsule [35,36]. Neurosarcoidosis and tuberculous meningitis are important differential diagnoses and may appear identical to meningovascular syphilis. Non-specific manifestations of meningovascular syphilis may include mild to moderate atrophy and white matter lesions [34]. This study noted cerebral atrophy in 64% of all patients. When CT and MRI studies of the brain favour a diagnosis of cerebral infarction, MR angiography is recommended to be the screening examination of choice in the diagnosis of vascular neurosyphilis because the arteritis more commonly affects large and medium-size vessels [33,34].

Two patients in this series (patients 3 and 13) manifested electroencephalographic changes. Hooshmand had reported EEG observations in 184/282 patients with neurosyphilis and noted abnormalities in 61.5% of records [37]. The two patients had Periodic lateralized epileptiform discharges (PLEDs), which did not resolve with intravenous lorazepam and therefore may not represent an ictal pattern. While PLEDs were initially reported in the setting of ischemic stroke, subsequently, these have been described in other acute destructive lesions of the cerebral cortex like focal encephalitis, traumatic brain injury, metastasis, among others, herpes simplex encephalitis [38]. Radhakrishnan et al. [39] and García-Morales et al. [40] had described the association of neurosyphilis with PLEDs and attributed PLEDs to underlying ischemia. In our study, the first patient had multiple cerebral infarctions, and the second one had Meningeal enhancement. Pathophysiologic mechanisms responsible for the periodicity in the EEG in the absence of ischemia or any other focal lesion are unknown but may reflect heightened neuronal excitability. PLEDs were reversible in 2 patients. This PLEDs particularity was reported in previous cases report [41]. Including neurosyphilis in the differential diagnosis of PLEDs should be stressed.

The treatment of syphilis has been proved controversial, and the best practice for management continues to be debated [42]. Penicillin remains the drug of choice for the treatment of syphilis. The CDC recommended two different regimens. The first one is aqueous crystalline penicillin G, administered intravenously every four hours for 10 to 14 days, in a dosage of 3 to 4 million units. The second regimen consists of penicillin G procaine, administered intramuscularly once daily in a dosage of 2.4 million units, plus probenecid, in a dosage of 500 mg orally four times daily, with both drugs given for 10 to 14 days [43]. For penicillin-allergic patients, an alternative regimen of ceftriaxone (2g/day IM or IV for 10-14 days) may be instituted, but this has not been as well tested for the management of neurosyphilis, and patients may have a cross-sensitivity to this agent [42]. This is the reason why some authors recommend penicillin desensitization for penicillin-allergic patients [43]. In our practice, we use crystalline penicillin G (30 million U IV daily) for 10 days, 3-monthly for one year. Follow-up of patients treated for neurosyphilis depends on the initial CSF findings. The CSF should be reexamined every six months if pleocytosis was present initially until the white blood cell count is normal. If the CSF white blood cell count does not decline after six months or completely normalize after two years, retreatment should be considered [43]. It is expected, within two years, that CSF parameters will normalize. Most treatment failures occur in immunocompromised patients [8,11].

Human immunodeficiency virus (HIV) and syphilis co-infection are common. The risk for neurosyphilis is increased in HIV patients. HIV testing should be systematic for all patients with syphilis, and regular screening for syphilis should be realized for all HIV-positive patients. Therefore, detection and treatment of syphilis can help to reduce HIV transmission. Syphilis may present with non-typical features in HIV-positive patients. There is a higher rate of non-symptomatic primary syphilis, and proportionately more HIV-positive patients present with secondary disease. Secondary infection may be more aggressive, and there is an increased rate of early neurological involvement [32]. Co-infection with HIV considerably complicates the interpretation of CSF abnormalities should be more complicated in syphilis patients coinfected with HIV because of increased protein, mononuclear pleocytosis, increased IgG, and the presence of oligoclonal bands may all attend HIV infection in the absence of NS [20]. In HIV-positive patients, relapse of the syphilis infection is more likely, so a careful follow-up is required.

## Conclusions

Meningovascular syphilis, although uncommon, is a treatable condition, particularly when it is diagnosed early. Clinical polymorphism seems to remain the hallmark of this disease. Based on the cases reviewed and similar reports in the literature, the combination of persistent and prominent stroke and headaches may suggest meningovascular syphilis even in the absence of meningism. Information about the current clinical spectrum of meningovascular syphilis is fundamental to maintain a high rate of suspicion worldwide and to promote the early diagnosis and treatment of a potentially devastating disease.
